# In vivo MRI assessment of bioactive magnetic iron oxide/human serum albumin nanoparticle delivery into the posterior segment of the eye in a rat model of retinal degeneration

**DOI:** 10.1186/s12951-018-0438-y

**Published:** 2019-01-10

**Authors:** Adi Tzameret, Hadas Ketter-Katz, Victoria Edelshtain, Ifat Sher, Enav Corem-Salkmon, Itay Levy, David Last, David Guez, Yael Mardor, Shlomo Margel, Ygal Rotenstrich

**Affiliations:** 1 Goldschleger Eye Institute, Sheba Medical Center, 52621 Tel-Hashomer, Israel; 2 Sackler Faculty of Medicine, Tel-Aviv University, 69978 Tel-Aviv, Israel; 3 Department of Chemistry, Bar-Ilan Institute of Nanotechnology and Advanced Materials, 52900 Ramat-Gan, Israel; 4 Advanced Technology Center, Sheba Medical Center, 52621 Ramat-Gan, Israel

**Keywords:** Iron oxide nanoparticles, RCS rats, Suprachoroidal injection, Retinal degeneration

## Abstract

**Background:**

Retinal degeneration diseases affect millions of patients worldwide and lead to incurable vision loss. These diseases are caused by pathologies in the retina and underlying choroid, located in the back of the eye. One of the major challenges in the development of treatments for these blinding diseases is the safe and efficient delivery of therapeutics into the back of the eye. Previous studies demonstrated that narrow size distribution core–shell near infra-red fluorescent iron oxide (IO) nanoparticles (NPs) coated with human serum albumin (HSA, IO/HSA NPs) increase the half-life of conjugated therapeutic factors, suggesting they may be used for sustained release of therapeutics. In the present study, the in vivo tracking by MRI and the long term safety of IO/HSA NPs delivery into the suprachoroid of a rat model of retinal degeneration were assessed.

**Results:**

Twenty-five Royal College of Surgeons (RCS) pigmented rats received suprachoroidal injection of 20-nm IO/HSA NPs into the right eye. The left eye was not injected and used as control. Animals were examined by magnetic resonance imaging (MRI), electroretinogram (ERG) and histology up to 30 weeks following injection. IO/HSA NPs were detected in the back part of the rats’ eyes up to 30 weeks following injection by MRI, and up to 6 weeks by histology. No significant differences in retinal structure and function were observed between injected and non-injected eyes. There was no significant difference in the weight of IO/HSA NP-injected animals compared to non-injected rats.

**Conclusions:**

MRI could track the nanoparticles in the posterior segment of the injected eyes demonstrating their long-term persistence, and highlighting the possible use of MRI for translational studies in animals and in future clinical studies. Suprachoroidal injection of IO/HSA NPs showed no sign of adverse effects on retinal structure and function in a rat model of retinal degeneration, suggesting that suprachoroidal delivery of IO/HSA NPs is safe and that these NPs may be used in future translational and clinical studies for extended release drug delivery at the back of the eye.

**Electronic supplementary material:**

The online version of this article (10.1186/s12951-018-0438-y) contains supplementary material, which is available to authorized users.

## Introduction

Retinal degeneration diseases affect millions of people worldwide and are currently untreatable. These include age-related macular degeneration (AMD) and diabetic retinopathy (DR) that are related to population aging and diabetes and are the leading cause of blindness and visual impairment worldwide [1, 2]. Retinitis pigmentosa (RP) is the most common genetically inherited retinal dystrophy affecting over 1 million patients [3]. These diseases are characterized by dysfunction of the neuro-retina and retinal pigmented epithelium (RPE)/choroid tissues, located in the posterior segment of the eye.

Treatment with neurotrophic or anti-angiogenic agents, may delay disease progression and presents a promising therapeutic approach [4–6]. However, the anatomy and physiology of the eye present a major challenge for safe and efficient drug delivery to the back of the eye. Topical eye drops are significantly impeded by the barrier of the corneal and conjunctival epithelium and rapid clearance by the tears, resulting in negligible penetration past the anterior segment of the eye [7]. Systemic treatments are limited by the blood–retina barrier and may also be associated with significant side-effects [8]. Intravitreal injections of corticosteroids and antibodies directed against vascular endothelial growth factor (VEGF) are the current clinical practice for treatment of neo-vascular AMD and DR [9, 10]. However, intravitreal drug delivery is limited by the isotropical drug diffusion through the vitreous towards other regions of the eye such as the ciliary body and lens and thus can lead to side effects including cataract, increased intraocular pressure (IOP) and glaucoma [11–13]. In addition, frequent repeated injections are required to overcome the drug elimination from the vitreous, that are not always well tolerated [14, 15]. Subretinal injection is an effective way of delivering treatments directly to the back of the eye. However it is a highly invasive procedure involving a complicated surgery with major side effects including damage to the retina. In addition, only small volumes can be injected using this method [16, 17]. Therefore, long-term delivery of therapeutics directly targeting the retina, RPE and choroid remains an unmet need [reviewed in 18].

We and others have shown that nanoparticles may be a promising drug delivery approach as they increase drug bioavailability and enable sustained release of bioactive molecules [19–22]. Specifically, superparamagnetic iron oxide (IO) NPs represent a promising drug delivery carrier as they are non-toxic and biodegradable [23, 24]. Furthermore, due to their iron content they can be tracked in vivo by magnetic resonance imaging (MRI) [25, 26]. In additions, different magnetic NPs have FDA approval for clinical use, mostly as MRI contrast agents for cancer patients (e.g., Endorem^®^, Resovist^®^ Combidex^®^) and treatment of iron deficiency (e.g. Feraheme^®^) [27].

In previous studies we described the generation of core–shell IO/human serum albumin (HSA) NPs for drug delivery [19]. HSA is a versatile protein carrier for drugs, it improves the pharmacokinetics of peptide or protein-based drugs, has low toxicity, it is readily available, biodegradable (an average blood half-life of 19 days) and has preferential uptake in tumors and inflamed tissues making it an ideal coating protein for NPs [28]. In addition, HSA bears various functional groups, (e.g., hydroxyls, amines, carboxylates and thiols), that can easily be used for conjugation of various biomolecules such as proteins and oligonucleotides [29, 30]. We have demonstrated that conjugation of the neuroprotective fibroblast growth factor 2 (FGF-2) to IO/HSA NPs reduces the growth factor susceptibility to enzymatic and thermal degradation, thus enhancing the protein biological efficacy [21]. These studies suggest that IO/HSA NPs may enable sustained release of the conjugated proteins.

The in vivo safety of intravitreal delivery of IO-NPs was demonstrated in several recent studies. Raju et al. demonstrated that intravitreal injection of 50 nm and 4 μm iron oxide nanoparticles, coated with either dextran polystyrene was non-toxic to the ocular structures [31]. Studies in *Xenopus* embryos also found no toxic effects following intravitreal injection of iron oxide NPs [20, 32, 33]. However, delivering the NPs into the vitreous may cause long term side effects related to the blocking of vision axis and off target effects due to diffusion of NPs in the vitreous that may reach other ocular tissues such as the trabecular meshwork [34].

The suprachoroidal space (SCS) is located between the choroid and sclera and its proximity to the choroid and RPE makes it an attractive site of drug delivery for retinal degeneration diseases. In addition, the SCS is not located in the vision axis and is relatively distant from the lens, anterior segment and ciliary body. Therefore, drug delivery to this compartment is predicted to result in significantly less off-target side effects. Our group has developed a new method for drug delivery into this compartment. In previous studies we demonstrated the long term safety and efficacy of delivering stem cells into the SCS in rats and rabbits in vivo [35, 36]. In addition, we demonstrated the efficacy and short term safety of delivering IO/HSA NPs into the SCS compartment of rabbits in vivo [37]. Immediately following injection, the IO/HSA NPs were spread across the extravascular matrix of the choroid, covering over 80% of the posterior eye surface. Histology analysis demonstrated that the injected IO/HSA NPs were retained in the choroidal extravascular matrix for at least 2 weeks following injection [37].

In the present study we evaluated the ability to track the injected NPs in vivo using MRI and assessed the long term safety of IO/HSA NP injection into the SCS of a rat model of retinal degeneration. IO/HSA NPs were injected into the SCS of Royal College of Surgeons (RCS) rats that are commonly used as an animal model in retinal degeneration translational studies. These rats carry a mutation in the gene encoding the MerTK receptor that mediates phagocytosis of the shed photoreceptor outer segments by the RPE cells. In the absence of functional MerTK, toxic outer segment debris accumulate in the subretina, forming a layer commonly termed the “debris zone”(DZ), and leading to photoreceptor cell death and vision loss [38–40]. A similar phenotype is observed in retinitis pigmentosa patients carrying mutations in this gene [40–42]. In the presented study we demonstrate the ability to track IO/HSA NPs in the posterior segment of RCS rat for up to 30 weeks by MRI and show no adverse effects on retinal structure or function up to 3 months following NP injection.

## Results

### MRI scanning

**Fig. 1:**
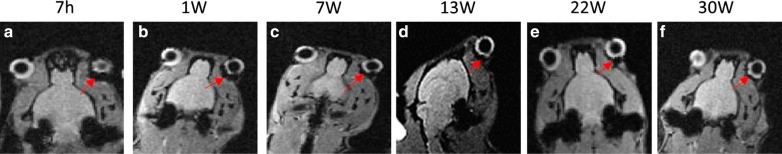
In vivo detection of IO/HSA NPs by MRI in a representative rat. The rat received a 5 µl suprachoroidal injection of IO/HSA NPs in the right eye. The left eye was not injected and was used as control. MRI was performed at 7 hours (**a**), and 1, 7, 13, 22 and 30 weeks (**b**–**f**) following injection. Red arrows highlight the hypointense areas on the T2* sequence at the back of the eye in the injected eye. *h* hours, *W* weeks

Ocular structures, lens and vitreous, were clearly demonstrated using T2* MRI scanning in the injected and non-injected eyes. In the injected eyes, the NPs could be observed as a dark, hypointense band underlying the entire back part of the injected eye up to 30 weeks following injection (Fig. 1 and Additional file 1: Figure S1). As shown in Additional file 2: Figure S2, the T1 and T2 scanning resulted in poorer detection of injected NPs compared to T2* imaging.

### Localization of injected IO/HSA NPs by Prussian Blue staining

**Fig. 2:**
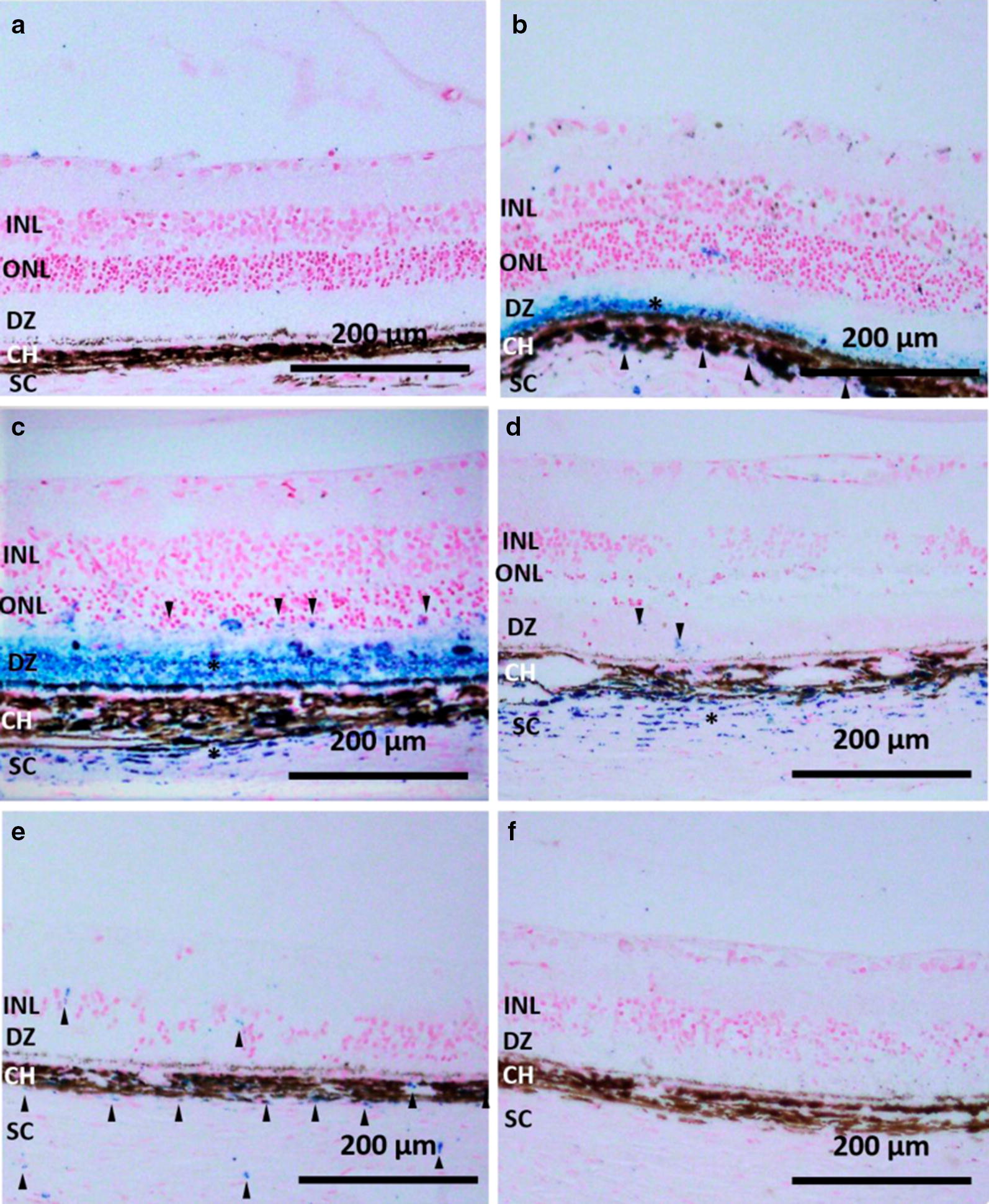
IO/HSA NPs localization in the RCS retina. Sections of RCS retinas removed 2 h (**b**),1 week (**c**), 4 weeks (**d**), 6 weeks (**e**), and 12 weeks (**f**) following injection as well as the contralateral non injected eye of the same rat shown in panel **b** (**a**) were stained with Prussian blue and counter stained with nuclear fast red. Asterisks highlight layers with positive Prussian blue staining, and arrowheads highlight focal Prussian blue staining. Scale bar 200 µm. *INL* inner nuclear layer, *ONL* outer nuclear layer, *DZ* debris zone, *CH* choroid, *SC* sclera

Next we examined the localization of injected NPs by Prussian Blue staining of retinal sections. As shown in Fig. 2, 2 h following injection, the IO/HSA NPs were found in the subretinal DZ layer, choroid and sclera (Fig. 2b). No staining was observed in the non-injected contralateral eyes in this and all other time points (Fig. 2a and data not shown). At 1-week post injection, positive Prussian Blue staining was clearly seen in the photoreceptor outer nuclear layer (ONL), the subretinal DZ, choroid and sclera (Fig. 2c). At 4 weeks following injection, the NPs were detected mainly in the choroid and sclera. Some scattered focal staining was demonstrated in the DZ (highlighted with black arrowheads in Fig. 2d). Six weeks post injection, the NPs were detected in the choroid and some scattered focal staining was demonstrated in the retina and sclera (highlighted with black arrowheads in Fig. 2e). No positive staining was observed in retinal sections obtained 12 weeks following NP injections (Fig. 2f).

### Histopathology analysis

**Fig. 3:**
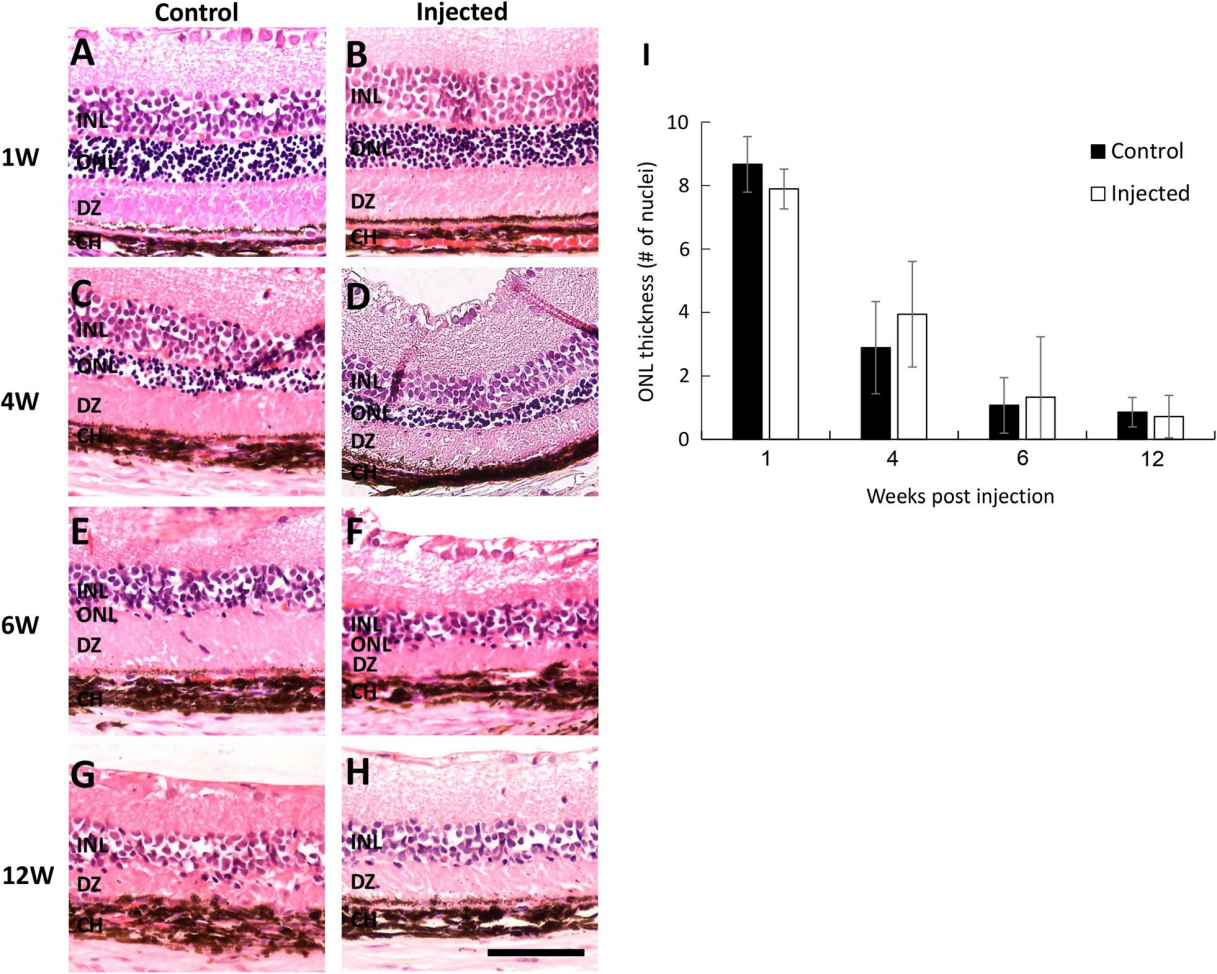
Histopathology analysis of RCS rat retinas. Representative images of retinal sections removed 1, 4, 6, and 12 weeks from non-injected (control, **A**, **C**, **E**, **G**) and contralateral injected eyes (**B**, **D**, **F**, **H**) stained with hematoxylin–eosin. Number of cell layers in ONL was evaluated by nuclei counting of 9 fields along the entire retina. Data is presented as mean ± SE from 2 to 4 eyes at each time point. Scale bar 100 μm

Gradual thinning of the outer nuclear layer in RCS rats due to photoreceptor degeneration has been extensively studied by our group and others [35, 38, 43]. As shown in Fig. 3, no significant changes in retinal structure were observed in the injected eyes compared with non-injected contralateral eyes. No scarring, bleeding, retinal detachment or inflammation were observed at any time point following NP injection. There were no significant differences in ONL thickness between injected and non-injected contralateral eyes at any time point following injection (Fig. 3I, all p > 0.212), suggesting that NP injection had no adverse effect on retinal structure.

### ERG recording

**Fig. 4:**
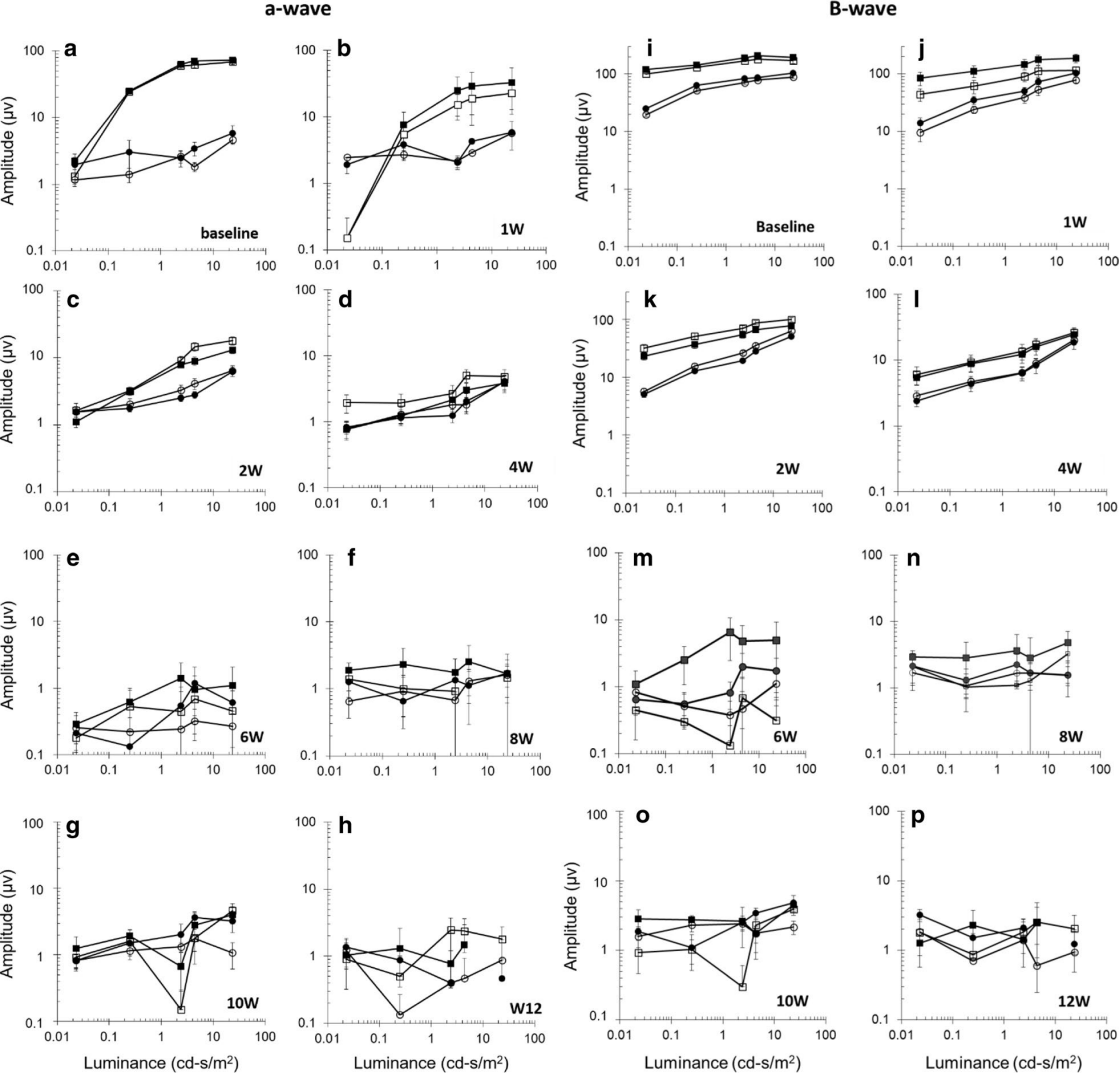
Electroretinogram recording showing retinal function in RCS rats following IO/HSA NP suprachoroidal injection. Dark-(squares) and light-(circles) adapted a-wave (**a**–**h**) and b-wave (**i**–**p**) amplitudes recorded in the injected (closed) and contralateral non injected control (open) eyes in the indicated time points following NP injection. Data are presented as mean ± SE

To examine whether suprachoroidal injection of IO/HSA NPs effected RCS retinal function, rats were tested for full-field ERG that measures the mass electrical response of the retina to light stimuli and is commonly used in the clinic and in translational studies for objective assessment of retinal function. Specifically, we determined the amplitudes of a- and b-waves that reflect the function of photoreceptors and bipolar cells, respectively [44, 45]. We and others have shown that ERG a- and b-wave amplitudes gradually diminish in RCS rats with age as photoreceptors degenerate [35, 43, 46, 47]. As shown in Fig. 4, no significant differences were observed between a-wave and b-wave amplitudes recorded in injected and control non-injected contralateral eyes in all time points following injection (all p values > 0.1).

### Weight

**Fig. 5:**
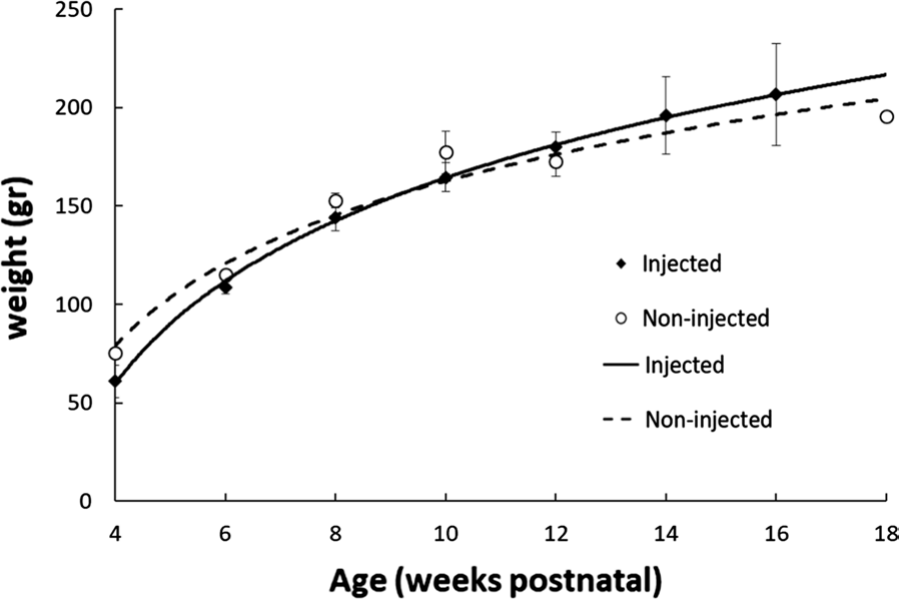
No significant differences were found in the body weight of injected and non-injected RCS rats at the same ages. Data is presented as mean ± SE with logarithmic regression lines

Animals were monitored for general health by weekly observation. No significant differences were found in animal body weight at any time point following NP injection in comparison with non-injected rats (all p > 0.090, Fig. 5).

## Discussion

In this study we demonstrated the long term tracking of IO/HSA NPs in the posterior segment of RCS rat eyes by MRI as well as the safety of suprachoroidal injection of IO/HSA NPs in close proximity to the choroid, RPE and photoreceptor cell layers. Since these are the target tissues affected in retinal degeneration diseases, our study suggests that IO/HAS NPs injected into the suprachoroidal space may present a potential novel drug delivery system for treatment of incurable blinding retinal degeneration diseases.

MRI detected the IO/HSA NPs in the back of the eye up to 30 weeks following injection (Fig. 1, Additional file 1: Figure S1), suggesting the potential use of IO/HSA NPs injected into the suprachoroidal space for long sustained drug release at the desired location of the posterior segment. By contrast, when 50 nm diameter superparamagnetic dextran-coated nanoparticles were injected into the vitreous of Sprague–Dawley rats, they could be detected by MRI only up to 1 week following injection [48]. In addition, in our study, MRI scanning demonstrated the spreading of the IO/HSA NPs in the posterior segment, covering most of the back of the eye surface, whereas in the Raju et al. study, the intravitreal injected superparamagnetic NPs were localized in a limited area of the back of the eye [48]. The entire retina is affected in retinitis pigmentosa [3]. Recent studies suggest that AMD patients have peripheral retinal changes including drusen accumulation and pigment changes associated with delayed dark adaptation [49, 50], emphasizing the need to deliver the therapeutics to the entire retina in these diseases. Our data suggest that the novel suprachoroidal IO/HSA NPs injection may present a promising intraocular drug delivery approach to achieve distribution of the treatment throughout the posterior segment by a single injection with prolonged intraocular residence. Furthermore, as RP, AMD and DR are chronic diseases, our data suggest that suprachoroidal delivery of IO/HSA NPs may enable sustained drug release at the posterior segment. Future translational studies will assess the safety and efficacy of drugs coupled to the IO/HSA NPs delivered to this compartment.

The aim of the presented study was to determine the safety, distribution and tissue persistence of IO/HSA NPs injected into the suprachoroidal space of rat eyes. The injection volume (5 μl) was chosen based on our previous studies that demonstrated the safety and efficacy of stem cell injection into this compartment [35]. The human retinal surface is ~ 100 times larger than the rat’s retina. In the clinic, drugs are injected into the vitreous of patients using a volume of 50 μl, which is 10-fold lower than the relative volume that was injected into the rats’ suprachoroid. The presented study demonstrates the safety of NP delivery in this volume, suggesting that this volume may be clinically relevant for delivery of therapeutics into this posterior segment compartment.

As we aimed at testing the safety of the IO/HSA NP delivery, a very high concentration of NPs was used (7.5 mg/ml). Our data suggest no toxicity effect in response of suprachoroidal injection of this high dose. Future studies will be aimed at testing the safety and efficacy of delivering therapeutics bound to these IO/HSA NP as well as determination of sensitivity of MRI detection of IONPS at different doses.

Several studies suggest that surface functionalization can enhance NP targeting to certain ocular tissues. For example hyaluronan (HA)-modified core–shell liponanoparticles increased RPE targeting following intravitreal injection of the NPs in rats through the interaction between the CD44 receptor on RPE cells and the HA ligand [51]. Coating iron oxide NPs with vascular endothelial growth factor (VEGF), enhanced NP concentration in the vascular choroid tissue following intravitreal injection in zebrafish embryo, most likely by binding VEGF receptor expressed in choroidal endothelial cells [33]. Our data demonstrates that the suprachoroidal injection directly targets the NPs to the subretina, choroid and sclera. It remains to be determined whether surface functionalization can further narrow NP targeting to a specific posterior segment tissue and enhance their persistence at that tissue.

Prussian blue staining demonstrated the localization of IO/HSA NPs in the subretina, choroid and sclera, up to 1 month following suprachoroidal injection and in the choroid and sclera up to 6 weeks following injection (Fig. 2). The findings that no positive staining was obtained in retinal sections removed at 12 weeks post injection whereas MRI scanning detected a hypointense area at the back of the injected eyes up to 30 weeks post injection, suggest that MRI is a more sensitive method for the detection of injected IO/HSA NPs than Prussian blue staining. Both MRI and Prussian blue staining failed to detect IO/HSA NPs in the non-injected contralateral eye at all time points following injection. These data suggest that even though the NPs were injected in close proximity to the choroidal vessels, they were retained in the ocular posterior segment for a long duration and upon clearance did not accumulate in the contralateral eye at a detectable level. The clearance mechanism of the NPs from the eye is unknown and requires further investigation. Nevertheless, general heath and weight monitoring suggested no systemic adverse effects of the suprachoroidal delivery of the IO/HSA NPs.

Deposition of iron in the retina (siderosis) exerts a toxic effect on cellular enzyme systems that results in RPE and retinal cell death. ERG is a very sensitive method to detect iron-related retinal damage [52–54]. Our ERG and histology data suggest no adverse effects of the suprachoroidal injection of the IO/HSA NPs on retinal function and structure, confirming the safety of this system. Of note, the ERG recordings and histology data obtained in the injected rats are in accordance with previous studies in RCS rats by our group [35, 43] and by others [55–58]. Our findings are consistent with other studies using coated NPs demonstrating lack of toxicity of intravitreal injected coated iron oxide NPs in several species [20, 31]. In previous studies we demonstrated the increased half-life and biological activity of FGF-2 coupled to the IO/HSA NPs in vitro in mesenchymal stromal cell cultures [21]. Future studies will be aimed at testing the potency of treating RCS rats and other rodent models of retinal degeneration with suprachoroidal injected IO/HSA NPs coupled with neurotrophic factors and other drugs in vivo. In addition, further studies are required to determine the effect of NP coating and surface functionalization on their clearance from the eye and bio-distribution in the eye tissues.

## Conclusions

Our data demonstrate the long-term detection of IO/HSA NPs in vivo in the posterior segment of RCS rats by MRI and the safety of suprachoroidal injection of these NPs. IO/HSA NPs may potentially be used for extended release drug delivery in the posterior segment. MRI may be used for translational studies in animals and in future clinical studies for assessment of IO/HSA NP bio-distribution.

## Methods

### Animals

Twenty-five Royal College of Surgeons (RCS) pigmented rats were used in this study. Rats were born and bred in the animal facility at Sheba Medical Center. Animals were kept under dim cyclic light (12 h in darkness and 12 h at < 5 lx). All animal experiments were conducted under the supervision and approval of the Institutional Animal Care Committee at the Sheba Medical Center, Tel-Hashomer. All procedures were conducted according to the recommendations of the Association for Research in Vision and Ophthalmology Statement for the Use of Animals in Ophthalmic and Vision Research.

### Preparation of IO/HSA NPs

The nanoparticles used in this study are superparamagnetic NPs with an iron oxide (IO) core and human serum albumin (HAS) coating. These IO/HSA NPs have one narrow size population with dry diameter of 21 ± 3 nm and hydrodynamic diameter 43 ± 5 nm. The NPs were prepared by nucleation followed by stepwise controlled growth of iron oxide (IO) thin films onto gelatin covalently conjugated with NHS Cy7 to obtain near infra-red (NIR)-IO/HSA NPs, as we previously described [21].

### Suprachoroidal injection of the IO/HSA NPs

The NPs were injected to the supra-choroidal space of 25 RCS rats at the age of 28 days postnatal. Five microliters of NP solution (7.5 mg/ml in PBS) were injected into the suprachoroid of the right eye under a surgical microscope (Leica Wild M690; Wild Herring, Herring, Switzerland) using our previously described protocol [35]. Rats were under intraperitoneal anesthesia of xylazine (10 mg/kg) and ketamine (75 mg/kg). The left eye was not injected and was used as control in all rats except two rats (rats #2 and #3, Additional file 1: Figure S1) which received an injection of NPs in both eyes.

### MRI scanning

In vivo tracking of injected NPs was performed by MRI in 7 rats. MRI scans were performed 7 h and 1, 7, 13, 22 and 30 weeks following injection (n ≥ 5 eyes in each time point). The rats were scanned under full xylazine and ketamine anesthesia as described above, using a clinical 1.5 T GE MR system (Optima MR450w, GE Healthcare, Chicago, Illinois, USA). The rats were placed in human phased array wrist coil and scanned using the following sequences:

1. Coronal Fast Spin Echo T1-weighted MRI (with relatively low sensitivity to iron oxide) with the following parameters: Field of view of 10 × 10 cm, slice thickness of 1 mm, matrix = 256 × 224 pixels, echo time of 13.63 ms, repetition time of 611 ms, 2 echo trains, bandwidth of 15.63 kHz and 4 repetitive acquisitions. Total scan time was 4′36″ for 16 slices.
2. Coronal Fast Spin Echo T2-weighted MRI (with relatively higher sensitivity to iron oxide) with the following parameters: Field of view of 10 × 10 cm, slice thickness of 1 mm, matrix = 256 × 224 pixels, echo time of 85 ms, repetition time of 5548 ms, 18 echo trains, bandwidth of 20.83 kHz and 4 repetitive acquisitions. Total scan time was 5′16″ for 16 slices.
3. Coronal Gradient Echo T2*-weighted MRI (with relatively high sensitivity to iron oxide) with the following parameters: Field of view of 10 × 10 cm, slice thickness of 1 mm, matrix = 256 × 224 pixels, echo time of 13 ms, repetition time of 300 ms, bandwidth of 15.63 kHz and 2 repetitive acquisitions. Total scan time was 4′36″ for 16 slices. The T2*-weighted gradient echo sequence is a highly sensitive sequence to detect the susceptibility artifacts (hypointensities) generated by iron oxide NPs [26].

### Histology

Fifteen animals were sacrificed at different time points following injection (1 h: n = 1; 1 week: n = 2, 4 weeks: n = 2; 6 weeks: n = 6; 12 weeks: n = 4) and eyes were removed for histology analysis. Eyes were fixed in formalin, embedded in paraffin and 4 µm sections were cut along the vertical meridian of the eye through the optic nerve as we previously described [35]. Sections were stained with Hematoxylin and eosin or Prussian Blue iron stain and Nuclear Fast Red as we previously described [21, 37]. Sections were visualized and photographed by light microscopy (Olympus BX51).

### Electroretinogram (ERG) recording

ERG recording was performed prior to injection and every 2 weeks following injection. ERG procedure was performed as we previously described [35, 43]. Briefly, the ERG was recorded using UTAS ERG system with a BigShot Ganzfeld (LKC, Technologies, Inc.) stimulator. The rats were placed inside the Ganzfeld bowl after 16 h dark adaptation. Golden wire loops were placed on the corneas and ERG signals were recorded from both eyes simultaneously. A reference chloride silver electrode was subcutaneously placed near the temporal canthus and the ground electrode was placed on the tail. Responses were amplified at 10,000 gain at 0.1–1000 Hz, filtered to remove 60 Hz noise, and digitized at a 10-kHz rate. For dark-adapted ERG, responses were averaged with stimulus intervals of 1–30 s depending on the stimulus luminance level. For light adapted ERG, the animals were light-adapted for 10 min prior to testing and responses were averaged with stimulus intervals of 1 s.

### Weight monitoring

The rats were weighed every 2 weeks and were monitored for general health during experiments. Weights were compared with the weight of 40 non-injected RCS rats from our laboratory data base.

### Statistical analysis

MANOVA analysis was performed to evaluate the effect of NP injection on ERG. Data consisted 8 weeks, 5 light intensities and two eyes (injected versus control). Time in weeks and light intensity were defined as within-subjects factors while the injected eye versus the control eye were defined as between-subjects factor. ERG amplitude recordings were the dependent variables. We performed separate MANOVA tests for each ERG wave type (b and a wave) and for each light adaptation conditions (photopic and scotopic adaptation). Kruskal–Wallis test was preformed to evaluate the difference in body weight of injected and non-injected RCS rats. One way ANOVA was preformed to evaluate the differences in ONL thickness in the injected eye and non-injected eyes in RCS rats at different time points following injection. The number of ONL nuclei in the time point of 1 week following injection, was not normally distributed. Hence the difference between injected and non-injected eyes was analyzed with Kruskal–Wallis test. All analyses were performed using SPSS 20.0 for Windows. Differences were considered significant if p < 0.05.

## Additional files

**Additional file 1: Figure S1.** MRI detection of injected IO/HSA NPs. Rats received a 5 µl suprachoroidal injection of IO/HSA NPs. Rats #2 and #3 were injected in the right (RE) and left (LE) eyes, as indicated on the left. Rats #1, #4 and #5 were injected only in the right eye (RE) and the left eye and was not injected, as marked. MRI was performed at indicated time points following NP injection. Red arrows highlight the hypointense areas on the T2* sequence at the back of the eye in the injected eyes. In some cases two scans are presented for each rat to demonstrate both eyes. *h* hours, *W* weeks.

**Additional file 2: Figure S1.** MRI scanning using the 3 protocols in a representative rat (rat #1 shown in Fig. 1). Red arrows highlight the hypointense areas at the back of the eye in the injected eye. The T2* sequence was more sensitive to the iron oxide disturbance of the magnetic field than the T1 and T2 scanning methods.

## Abbreviations

AMD: age-related macular degeneration
ANOVA: analysis of variance
CH: choroid
DZ: debris zone
DR: diabetic retinopathy
ERG: electroretinogram
FGF-2: fibroblast growth factor 2
FDA: Food and Drug Administration
HA: hyaluronan
INL: inner nuclear layer
IOP: intraocular pressure
IO/HSA NPs: iron oxide/human serum albumin nanoparticles
MRI: magnetic resonance imaging
MANOVA: multivariate analysis of variance
NIR-IONPs: near infra-red-iron oxide nanoparticles
ONL: outer nuclear layer
PBS: phosphate buffered saline
RCS: Royal College of Surgeons
RPE: retinal pigmented epithelium
RP: retinitis pigmentosa
SC: sclera
SE: standard error
SCS: suprachoroidal space
VEGF: vascular endothelial growth factor
