# Supplementary figures and images for: In vivo MRI assessment of bioactive magnetic iron oxide/human serum albumin nanoparticle delivery into the posterior segment of the eye in a rat model of retinal degeneration
Source: J Nanobiotechnology. 2019 Jan 10;17:3. doi: 10.1186/s12951-018-0438-y (PMC6327435; doi:10.1186/s12951-018-0438-y)

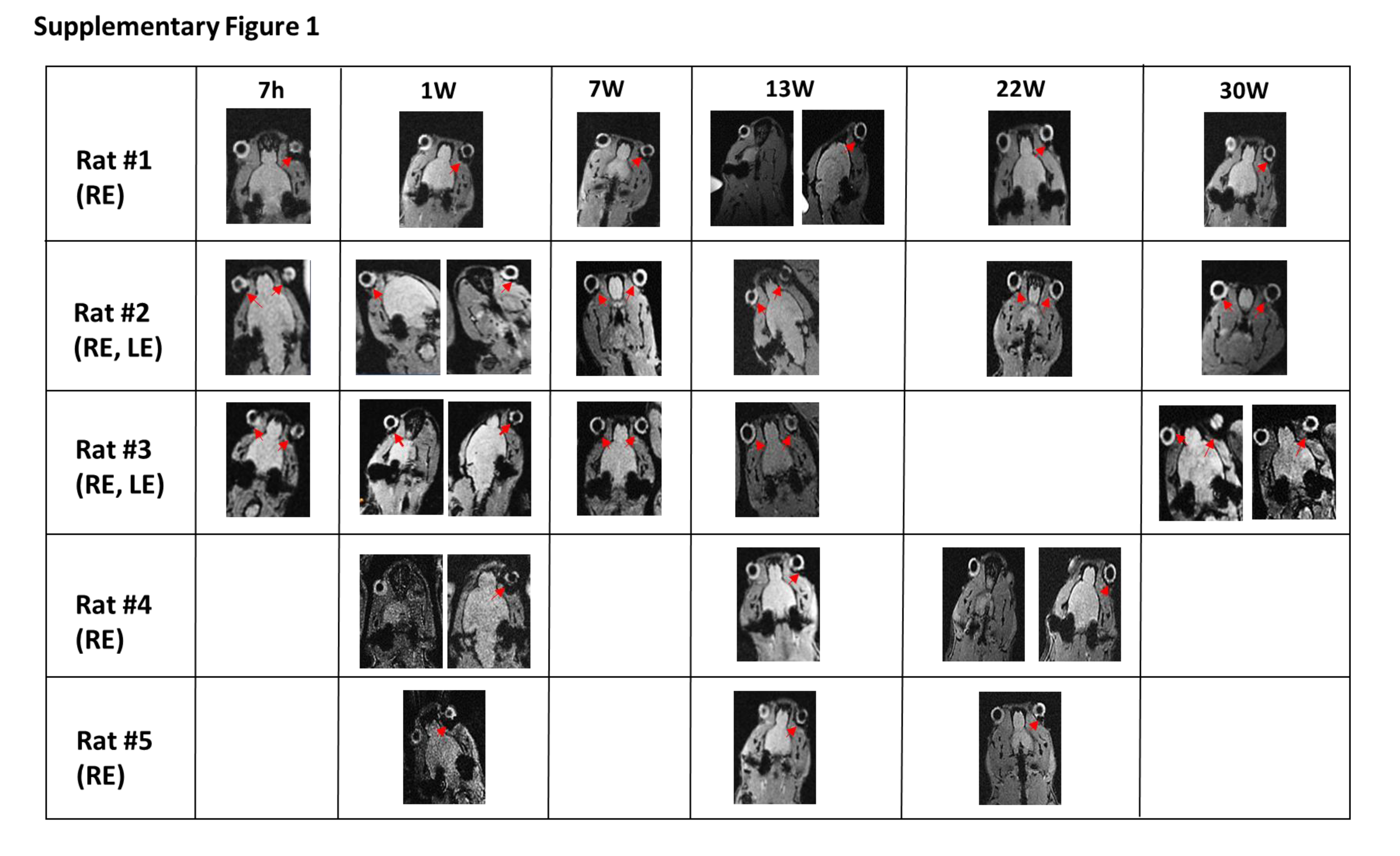

Supplement: Supplementary file 1 — Additional file 1: Figure S1. MRI detection of injected IO/HSA NPs. Rats received a 5 µl suprachoroidal injection of IO/HSA NPs. Rats #2 and #3 were injected in the right (RE) and left (LE) eyes, as indicated on the left. Rats #1, #4 and #5 were injected only in the right eye (RE) and the left eye and was not injected, as marked. MRI was performed at indicated time points following NP injection. Red arrows highlight the hypointense areas on the T2* sequence at the back of the eye in the injected eyes. In some cases two scans are presented for each rat to demonstrate both eyes. h hours, W weeks. [file 12951_2018_438_MOESM1_ESM.tif]

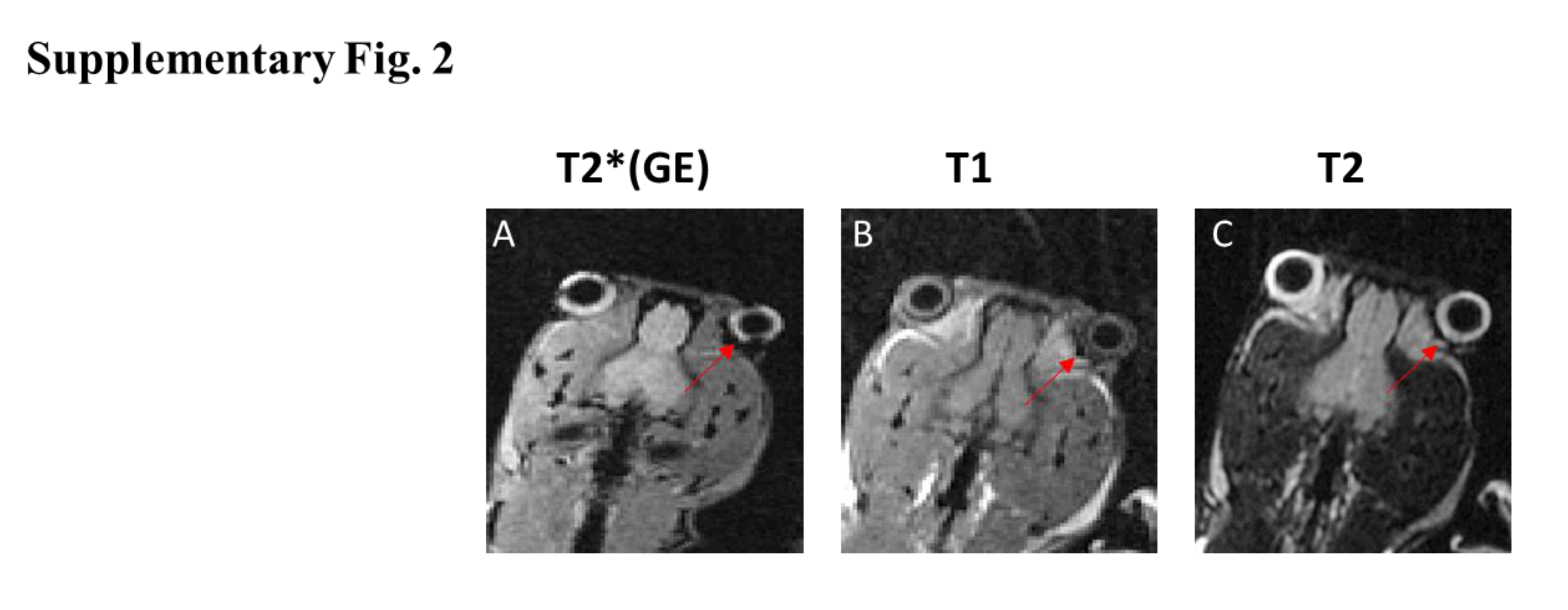

Supplement: Supplementary file 2 — Additional file 2: Figure S1. MRI scanning using the 3 protocols in a representative rat (rat #1 shown in Fig. 1). Red arrows highlight the hypointense areas at the back of the eye in the injected eye. The T2* sequence was more sensitive to the iron oxide disturbance of the magnetic field than the T1 and T2 scanning methods. [file 12951_2018_438_MOESM2_ESM.tif]
